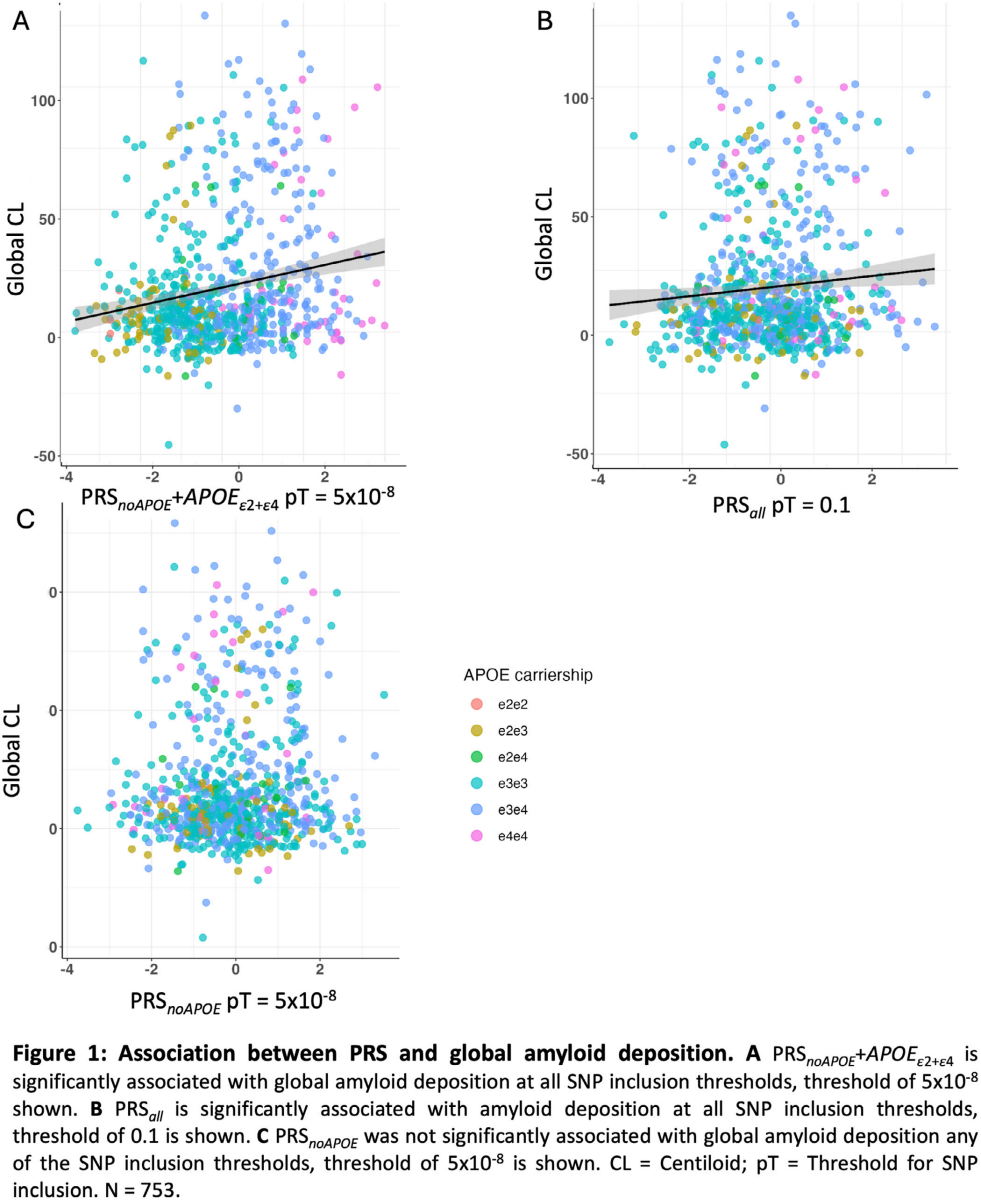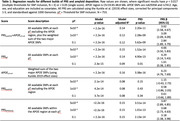# Associations between AD polygenic risk scores and global amyloid deposition in the European AMYPAD consortium

**DOI:** 10.1002/alz.088788

**Published:** 2025-01-03

**Authors:** Emma S. Luckett, Yasmina Abakkouy, Lyduine E. Collij, David Vállez García, Isadora Lopes Alves, Luigi Lorenzini, Juan Domingo Gispert, Pieter Jelle Visser, Anouk den Braber, Craig W Ritchie, Rik Vandenberghe, Robin Wolz, Mahnaz Shekari, Natalia Vilor‐Tejedor, Gill Farrar, Frederik Barkhof, Isabelle Cleynen, the AMYPAD consortium

**Affiliations:** ^1^ Laboratory for Cognitive Neurology, Leuven Brain Institute, KU Leuven, Leuven Belgium; ^2^ Karolinska Institutet, Stockholm Sweden; ^3^ Laboratory for Complex Genetics, KU Leuven, Leuven Belgium; ^4^ Amsterdam UMC, location VUmc, Amsterdam Netherlands; ^5^ Lund University, Lund Sweden; ^6^ Amsterdam UMC, Amsterdam Netherlands; ^7^ Brain Research Center, Amsterdam Netherlands; ^8^ Universitat Pompeu Fabra, Barcelona Spain; ^9^ Barcelonaβeta Brain Research Center (BBRC), Pasqual Maragall Foundation, Barcelona Spain; ^10^ Centro de Investigación Biomédica en Red Bioingeniería, Biomateriales y Nanomedicina, Madrid Spain; ^11^ Alzheimer Center Amsterdam, Neurology, Vrije Universiteit Amsterdam, Amsterdam UMC location VUmc, Amsterdam Netherlands; ^12^ Biological Psychiatry, Vrije Universiteit Amsterdam, Amsterdam Netherlands; ^13^ Scottish Brain Sciences, Edinburgh United Kingdom; ^14^ Neurology Department, University Hospitals Leuven, Leuven Belgium; ^15^ IXICO, London, Greater London United Kingdom; ^16^ IMIM (Hospital del Mar Medical Research Institute), Barcelona Spain; ^17^ Department of Clinical Genetics, Erasmus University Medical Center, Rotterdam Netherlands; ^18^ Hospital del Mar Research Institute (IMIM), Barcelona Spain; ^19^ Barcelonaβeta Brain Research Center (BBRC), Barcelona Spain; ^20^ Centre for Genomic Regulation (CRG), Barcelona Institute of Science and Technology (BIST), Barcelona Spain; ^21^ GE HealthCare, Amersham United Kingdom; ^22^ Department of Radiology and Nuclear Medicine, Amsterdam Neuroscience, Vrije Universiteit Amsterdam, Amsterdam UMC, Amsterdam Netherlands; ^23^ Queen Square Institute of Neurology and Centre for Medical Image Computing, UCL, London Kingdom United; ^24^ The project leading to this paper has received funding from the Innovative Medicines Initiative 2 Joint Undertaking under grant agreement No 115952, Brussels Belgium

## Abstract

**Background:**

Published data have highlighted associations between Alzheimer’s disease (AD) susceptibility loci and AD‐related brain changes. The amyloid imaging to prevent AD (AMYPAD) consortium is a European collaboration consisting of several parent cohorts, four of which had raw genotype array data available. We sought to integrate and harmonise the genetic data, calculate AD polygenic risk scores (PRS), and investigate their association with global amyloid deposition.

**Method:**

Raw genetic data (GRCh37) was available for 753 non‐demented participants, which underwent standard pre‐imputation quality control (QC) using PLINK. Imputation was performed using the Michigan Imputation server and HRC reference panel, and standard post‐imputation QC was done prior to the final merging of cohorts. PRSice was used for PRS calculations with Stage 1 summary statistics from Kunkle et al. (2019) as base file and European individuals from 1000 Genomes as reference for clumping. We calculated several builds of PRS at three *p*‐value thresholds for SNP inclusion: 5 × 10^‐8^, 1 × 10^‐5^, and 0.1. These included PRS*_all_*, PRS*_APOEonly_* (Chr19:45‐48.8Mb), *APOE_ε_*_2+_ *_ε_*_4_ (the weighted sum of the two major *APOE* SNPs rs429358 and rs7412), PRS*_noAPOE_* (all SNPs excluding Chr19:45‐48.8Mb), and a composite PRS*_noAPOE_* +*APOE_ε_*_2+_ *_ε_*_4_. All were corrected for genetic principal components 1‐5 and standardised against 1000 Genomes. Cross‐sectional global amyloid burden (expressed in Centiloids) was available for everyone. Linear regression models determined if PRS were associated with amyloid deposition (age, sex, and education as covariates). Significance was based on an uncorrected *p*<0.05 divided by the number of SNP inclusion thresholds (N = 3).

**Result:**

Participants were 66.9±7.6 years, 58.6% female, and 207 (27%) considered amyloid positive (CL>21). In this preliminary analysis, we found that most PRS builds were significantly associated with amyloid at all SNP inclusion thresholds assessed (Table 1, representative plots Fig. 1A,1B), except for PRS*_noAPOE_* (Table 1, representative plot Fig. 1C).

**Conclusion:**

We highlight the significant influence of *APOE* in determining AD genetic predisposition, and that AD PRS are associated with amyloid deposition. Our results show that AMYPAD is a suitable cohort for studying genetic factors driving amyloid deposition before clinical onset, which we aim to extend with the inclusion of more parent cohorts, and regional and longitudinal amyloid data.